# Comprehensive Diagnosis of Bacterial Infection Associated with Acute Cholecystitis Using Metagenomic Approach

**DOI:** 10.3389/fmicb.2017.00685

**Published:** 2017-04-20

**Authors:** Manabu Kujiraoka, Makoto Kuroda, Koji Asai, Tsuyoshi Sekizuka, Kengo Kato, Manabu Watanabe, Hiroshi Matsukiyo, Tomoaki Saito, Tomotaka Ishii, Natsuya Katada, Yoshihisa Saida, Shinya Kusachi

**Affiliations:** ^1^Department of Surgery, Toho University Ohashi Medical CenterTokyo, Japan; ^2^Laboratory of Bacterial Genomics, Pathogen Genomics Center, National Institute of Infectious DiseasesTokyo, Japan

**Keywords:** acute cholecystitis, metagenome analysis, next-generation sequencing, bacteriological analysis, gut microbiota

## Abstract

Acute cholecystitis (AC), which is strongly associated with retrograde bacterial infection, is an inflammatory disease that can be fatal if inappropriately treated. Currently, bacterial culture testing, which is basically recommended to detect the etiological agent, is a time-consuming (4–6 days), non-comprehensive approach. To rapidly detect a potential pathogen and predict its antimicrobial susceptibility, we undertook a metagenomic approach to characterize the bacterial infection associated with AC. Six patients (P1–P6) who underwent cholecystectomy for AC were enrolled in this study. Metagenome analysis demonstrated possible single or multiple bacterial infections in four patients (P1, P2, P3, and P4) with 24-h experimental procedures; in addition, the CTX-M extended-spectrum ß-lactamase (ESBL) gene was identified in two bile samples (P1 and P4). Further whole genome sequencing of *Escherichia coli* isolates suggested that CTX-M-27-producing ST131 and CTX-M-14-producing novel-ST were identified in P1 and P4, respectively. Metagenome analysis of feces and saliva also suggested some imbalance in the microbiota for more comprehensive assessment of patients with AC. In conclusion, metagenome analysis was useful for rapid bacterial diagnostics, including assessing potential antimicrobial susceptibility, in patients with AC.

## Introduction

Acute cholecystitis (AC) is the acute inflammation of the gallbladder. AC is usually associated with cholelithiasis, with gallstones detected in >90% cases. Additional causes include circulation defect of the gallbladder wall, bacterial infection, and chemical disorder (van der Linden and Sunzel, 1970; Wang et al., 2016). The Tokyo Guidelines 07, published in 2007 following the international consensus meeting on the management of AC and cholangitis, were revised in 2013 (TG13) (Takada et al., 2007, 2013). The guidelines recommend treatment based on the severity, with patients generally treated with antimicrobial agents before operation, drainage, or general care is considered. When conservative treatment with drainage is selected, antimicrobial agents are administered depending on the bile culture examination and antimicrobial susceptibility testing results.

Asai et al. reported that bactibilia was detected in 96 of 136 patients with AC (58.6%) (Asai et al., 2012), whereas Csendes et al. reported the rate, including that of cholelithiasis, was 22–46% (Csendes et al., 1996). The rate of bactibilia was reported to be 72% in TG13 (Takada et al., 2013); hence, the guidelines recommend bile culture for all cases. However, bile culture is a time-consuming, non-comprehensive approach that requires several days to obtain the results. Delay of appropriate treatment might result in a fatal condition and might increase antimicrobial resistance. Hence, the establishment of rapid diagnostics is important to determine an appropriate treatment. Furthermore, the microflora of the gut is believed to be associated with the prevention of infection and fatal condition (Wells et al., 2000; Wu et al., 2013; Liu et al., 2015). Currently, there are no reports regarding the use of a metagenomic approach to reveal comprehensive microbiota analysis of patients with AC.

Metagenome analysis by next-generation DNA sequencing (NGS) has led to a new method of identifying the etiological agents of an infectious disease (Tang and Chiu, 2010; Chan et al., 2012). By directly sequencing millions of DNA/RNA molecules in a specimen and matching the sequences to those in a database, pathogens can be inferred (Takeuchi et al., 2014). The conventional method requires bacteria to be individually cultured, whereas metagenome analysis is a comprehensive approach that enables the direct sequencing of bacterial DNA without bacterial cultivation (Takeuchi et al., 2014).

Here, we report the results of metagenome analysis of six cases (P1–P6) who underwent cholecystectomy for AC. This study was expected to provide additional information, such as bacterial genotyping and ratios of bacterial species compared with that provided by conventional examination. The risk factors of AC, which is associated with gut microbiota, may also become gradually clear with this approach. Moreover, the possibilities of obtaining rapid genotype analysis results of potential pathogens and also rapid evaluation of antimicrobial resistance are promising aspects of this technique.

## Materials and methods

### Patients

Between May 2015 and March 2016, six patients (P1–P6) who underwent cholecystectomy for AC at the Department of Surgery, Toho University Ohashi Medical Center were enrolled in this study. Written informed consent was obtained from the participants. The patients were diagnosed as having AC and were performed recommended antimicrobial treatment based on the TG13 severity grade during the waiting period for operation. The study protocol was approved by the ethics committee of the Toho University Ohashi medical Center (Approval No. 14-58, 11/18/2014) and National Institute of Infectious Diseases in Japan (Approval No. 642, 12/11/2015). It was conducted according to the principles of the Declaration of Helsinki.

### Materials

Bile samples were aseptically collected during the operation and divided into three tubes (two aerobic porters and one anaerobic porter, Kenki porter®). One aerobic and one anaerobic porter immediately underwent conventional culture and antimicrobial susceptibility testing at our hospital. The other aerobic porter was immediately frozen at −20°C for future metagenome analysis. In addition, the patients put saliva and feces into an aerobic porter during hospitalization, which was also immediately frozen at −20°C.

### Antimicrobial susceptibility testing

Antimicrobial susceptibility testing was performed using Neg EN Combo 1T® panels (Microscan Walkaway 96SI: Siemens) on the basis of the Clinical and Laboratory Standards Institute criteria (M100-S22) (Clinical and Laboratory Standards Institute, 2012). Extended-spectrum ß-lactamases (ESBLs) were assessed by a disk-diffusion test using cefotaxime, ceftazidime, and cefpodoxime with or without clavulanate.

### Metagenome analysis

The clinical specimens were mixed with EDTA (final 10 mM), SDS (final 1%), and proteinase K (final 100 μg/ml) and incubated at 50°C for 30 min. The samples were treated with an equal volume of 0.1-mm-diameter glass beads and lysed by bead beating using a vortex at a maximum speed for 10 min with a Genie2 vortex adapter (MO BIO). Following centrifugation to remove the debris, the supernatants were extracted using the QIAGEN QIAquick PCR Purification Kit. A DNA-seq library was prepared using the Illumina Nextra® XT DNA Sample Preparation Kit (Illumina; San Diego, CA, USA) and the indexing method. A deep sequencing run for single-end short reads (150-mer) using the MiSeq reagents kit v3 was performed using a MiSeq sequencer (Illumina; San Diego, CA, USA).

### Bioinformatics

To identify potential pathogens and detect antimicrobial resistance genes, sequencing reads were analyzed by the MePIC2 (Takeuchi et al., 2014), Krona (Ondov et al., 2011), and MEGAN6 software (Huson et al., 2007, 2016). To identify the bacterial sequence type and antimicrobial resistance, sequencing reads were analyzed by multilocus sequence typing (MLST) and Resfinder, respectively (Larsen et al., 2012; Zankari et al., 2012).

### Nucleotide sequence accession number

The metagenome analysis short-read sequences have been deposited in the DNA Data Bank of Japan (accession numbers: DRA005134).

## Results

The clinical presentations of all six patients are shown in Table 1. All patients underwent cholecystectomy for AC. The median age was 64.5 years (range: 43–85 years) and three were males and three were females. The causes of AC included gallbladder stone obstruction in four cases and unknown in two cases. The median time from onset to operation was 39.5 h, with the median time from admission to operation being 31 h. The patients underwent antimicrobial treatment several times before the operation, except for one case. Conventional bile culture examination results revealed that four cases (P1–P4) were bacteriologically positive and two (P5, P6) were negative (Table 1).

**Table 1:** **Clinical presentations and results of bacterial cultures**.

**Patient ID**	**P1**	**P2**	**P3**	**P4**	**P5**	**P6**
**CHARACTERISTICS OF PATIENTS**						
Age (years)	85	78	76	53	43	44
Sex (M/F)	F	M	M	F	M	F
Past medical history	Angina pectoris	Pleurisy	Lacunar infarction	Chronic pancreatitis	−	Cervical cancer
	Rheumatoid arthritis		Hypertension			
Cause of acute cholecystitis	Gallbladder stone	Gallbladder stone	Gallbladder stone	Gallbladder stone	−	−
**LABORATORY DATA**						
White blood cell count (cells/mm^3^)	10,500	14,200	10,400	5,600	12,600	3,100
C-reactive protein (mg/dL)	19.69	2.15	9.49	0.37	1.58	9.62
**PREOPERATIVE CHARACTERISTICS**						
Time from onset to operation (h)	55	41	46	6.4	33	53
Time from admission to operation (h)	22	41	42	0.4	20	39
Drainage	PTGBA	ERCP	NA	NA	NA	NA
Antimicrobial agents	CTRX	SBT/CPZ	SBT/CPZ	NA	SBT/CPZ	CTRX
Hospitalization (days)	22	10	10	3	4	7
**TG 13 SEVERITY CLASSIFICATION**						
Mild/Moderate/Severe	Mild	Mild	Moderate	Mild	Mild	Moderate
Bacterial profile of bile culture	*Escherichia coli* (ESBL)	*Escherichia coli*	*Escherichia coli*	*Escherichia coli* (ESBL)	Negative	Negative
			*Klebsiella pneumoniae*			
			*α-hemolytic Streptococcus*			

*M, Male; F, Female; PTGBA, Percutaneous transhepatic gallbladder aspiration; ERCP, Endoscopic retrograde Cholangiopancreatography; NA, Not applicable; TG13, Tokyo guidelines 2013; ESBL, Extended-spectrum ß-lactamase; CTRX, Ceftriaxone, SBT, Sulbactam, CPZ, Cefoperazone*.

**Figure 1:**
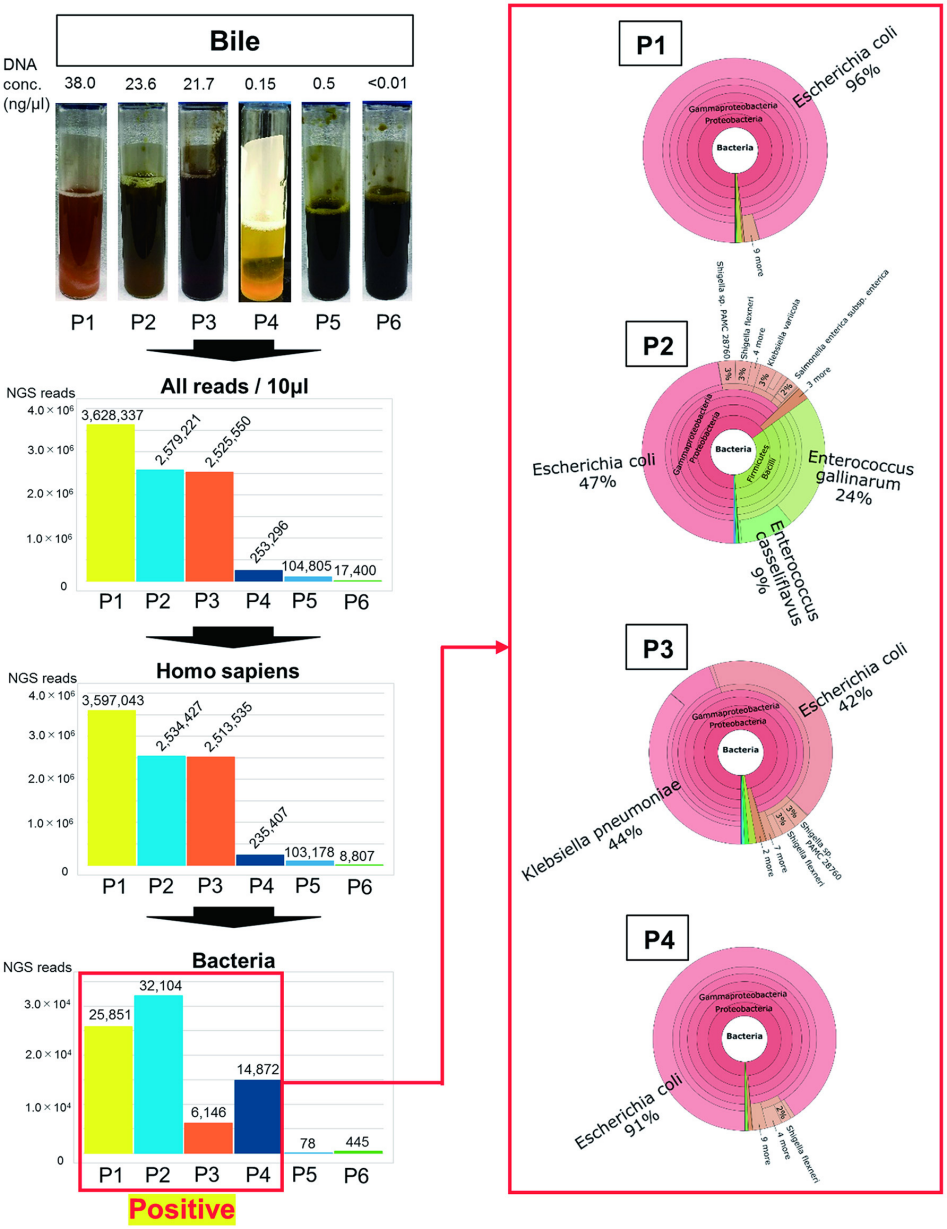
**Metagenome analysis of bile specimens**. The bile appearance was purulent (P1), mild cholestasis (P2), white (P4), and cholestasis (P3, P5, P6). From metagenome analysis, the ratio of *H. sapiens* DNA included in the specimens was high for bacteriologically positive cases. Furthermore, a high ratio of bacterial DNA was detected from samples that were bacteriologically positive by conventional culture examinations. The numbers on the bar graph represent the sequencing reads for each patient. Regarding the detected bacteria, the ratios of etiological agents of AC were rapidly identified within 24 h (shown in red box).

When evaluating mucous membrane inflammation, human DNA sequences were excluded from the MEPIC2 software analysis for ethical reasons, allowing for the detection of pathogens as shown in Figure 1. After this process, DNA derived from *Homo sapiens* was more abundant in bacteriologically positive cases compared with that in negative cases, suggesting an association between bacterial infection and exfoliation of the gallbladder mucous membrane. The ratio of bacterial DNA was also high in positive cases (Figure 1). Bacterial infection of the gallbladder was rapidly diagnosed by the metagenome approach (within 24 h) compared with that by the conventional approach (within 4–6 days). In feces and saliva samples, characteristic bacterial populations were identified, especially in the positive cases. Each case is described in detail.

### Patient 1 (P1)

Only ESBL-positive *E. coli* was detected in the bile by bacterial culture and antimicrobial susceptibility testing (Table 1). This patient had postoperative complications, such as an intraabdominal abscess.

As shown in Figure 1, the purulent bile was identified to be bacteriologically positive by the metagenomics approach. The ratio of bacterial DNA in the specimen was high, and the etiological agent was *E. coli* ST131 with the CTX-M-27 gene.

**Figure 2:**
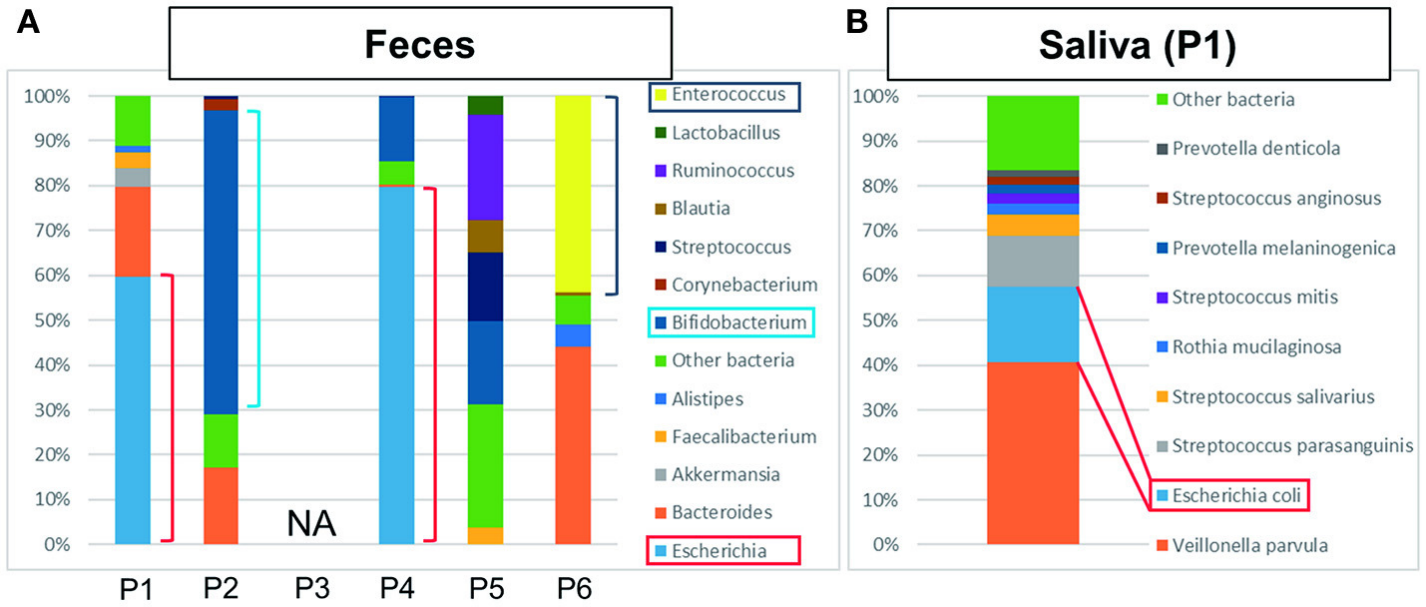
**The results of metagenome analysis of feces and saliva. (A)** P1–P4 were bacteriologically positive cases, P3 was not applicable (NA) and P5 and P6 were negative cases. In the cases of bactibilia, bacterial turbulence of feces was identified. Especially, a high ratio of *E. coli* was detected in P1 and P4 suggesting the AC was caused by *E. coli*. *Bifidobacterium* was detected at high ratio in P2. The bacterial flora in P5 was dominated by *Ruminococcus, Bifidobacterium, Streptococcus*, and “other bacteria.” In P6, dysbiosis, determined by the increased amount of *Enterococcus* detected, may be due to the influence of chemotherapy. **(B)** Characteristic saliva analysis shown for P1. A high ratio of *E. coli* was detected.

Bacterial detection from feces and saliva samples are shown in Figures 2A,B. Intriguingly, *E. coli* was detected at a high ratio in feces (60%) and saliva (17%). ST131 was also detected in both samples and also in the bile sample. Although *E. coli* was generally detected in a smaller percentage in feces and saliva (Becker et al., 2015), this result suggested that it was a marked etiological agent of AC and that dysbiosis may be related to postoperative complications.

### Patient 2 (P2)

During the treatment of pleurisy with levofloxacin, P2 developed AC because of cholelithiasis. LVFX-resistant *E. coli* was detected by conventional antimicrobial susceptibility testing; however, ESBL was not detected in the bile samples (Table 1). As shown in Figure 1, mild cholestasis was detected in the bile sample, and bactibilia was identified by metagenome analysis. The etiological agent was *E. coli*. Although, it is unclear whether *Enterococcus gallinarum* and *E. casseliflavus* were associated with AC, these were significantly detected by metagenome analysis.

*Bifidobacterium* was abundantly detected at 68% in feces (Figure 2A), which is higher than generally detected in a healthy gut (Odamaki et al., 2016). It was unknown whether P2 received probiotics.

### Patient 3 (P3)

In this case, *E. coli, K. pneumoniae*, and α*-hemolytic Streptococcus* were detected by the bacterial culture of bile, along with cholestasis (Table 1 and Figure 1). Metagenome analysis suggested that the bile sample was bacteriologically positive for *E. coli* and *K. pneumonia*, indicating a diagnosis of AC with multi-infection. We were unable to obtain saliva and feces samples from this patient.

### Patient 4 (P4)

ESBL-positive *E. coli* was detected by bile culture (Table 1). The appearance of bile was white, and it was bacteriologically positive by metagenome analysis (Figure 1). The etiological agent was *E. coli* that had the CTX-M-14 gene but with a novel sequence type. *E. coli* was detected at a high ratio (76%) in feces by metagenome analysis (Figure 2A). The microbiota of saliva is not significantly different from commensal oral microbiota, which is dominated by *Actinobacteria* and *Streptococcus* spp. (Lloyd-Price et al., 2016).

### Patient 5 (P5)

No bacteria were detected by bile culture in this case (Table 1). As shown in Figure 1, the appearance of bile was with cholestasis. Metagenome analysis was negative for bacterial infection, indicating that treatment with antimicrobial agents was not necessary. No significant differential flora was detected in feces (Figure 2A) and saliva. The bacteria detected in the feces appeared to be normal because it was dominated by *Ruminococcus* (23%), *Bifidobacterium* (18%), *Streptococcus* (15%), and “others” that included over two hundreds types of bacterial genera. A lesser amount of *Proteobacteria*, including *E. coli* (Qin et al., 2010; Becker et al., 2015), was detected.

### Patient 6 (P6)

The bile culture results were negative (Table 1). The appearance of bile was with cholestasis (Figure 1). Limited bacterial DNA was detected from the bile by metagenome analysis, similar to P5, suggesting bacteriologically negative samples. As for P5, it is not necessary to treat with antimicrobial agents. The ratio of bacterial species in the feces of the patient was different from that of healthy people, with *E. faecium* (44%) significantly detected. This result may be affected by chemotherapy.

The results of metagenome analysis were consistent with those of conventional culture examination and antimicrobial susceptibility testing for all six patients. Although qualitative evaluation was possible by conventional examination, quantitative evaluation was impossible. Metagenome analysis enables the quantitative evaluation. Metagenome analysis has no concern about missing the detection of potential pathogens with unculturable etiological agents.

In addition to the bile samples, feces and saliva samples were analyzed by metagenome approach to provide a more complete assessment of patients with AC. The bacteriologically positive cases showed some imbalance in the microbiota compared with negative cases.

## Discussion

NGS technologies are a new class of sequencing platforms, which differ from previous sequencing methods mainly based on Sanger sequencing, which have been developed and established over the last decade. These new instruments have revolutionized the field of genomics, providing enormous high-throughput and speed, with a wide range of applications now accessible to most laboratories. This technology has been applied to investigate the etiological agent of other inflammatory diseases, such as inflammatory bowel disease (IBD) (Morgan et al., 2012; Dalal and Chang, 2014). Furthermore, high-throughput sequencing has enabled the identification of previously uncharacterized viruses or bacteria as the etiological agents of infectious diseases (Tang and Chiu, 2010; Barzon et al., 2011; Chan et al., 2012; Lavezzo et al., 2013; Faria et al., 2016).

In this study, we analyzed six patients with AC using metagenome analysis with NGS. For the case of bactibilia, the metagenome analysis results were consistent with those of conventional culture examination and antimicrobial susceptibility testing.

A greater number of ESBL-positive *E. coli* NGS reads were detected by metagenome analysis in P1 and P4 compared with that in other patients. Several reports have been published on the gut microbiota in patients with IBD showing an increase in the number of species belonging to *Proteobacteria*, including *E. coli* (Frank et al., 2007; Morgan et al., 2012). The proportion of *Proteobacteria* in healthy human intestinal microbiota was shown to be 1% (Tap et al., 2009). AC for P1, who had more *E. coli* in the feces sample compared with healthy people, was most likely caused by *E. coli*. Furthermore, *E. coli* was more significantly detected in P1 without vomiting than in healthy people (Clemente et al., 2012). In this case, the postoperative complication of an intraabdominal abscess was detected, prolonging the duration of hospitalization compared with that in other patients (Table 1).

*E. coli* ST131, which is a particularly virulent strain of *E. coli*, was first described in (Nicolas-Chanoine et al., 2008). The dissemination has been globally reported, both in the healthcare and community settings (Rogers et al., 2011; Nicolas-Chanoine et al., 2014), and is mostly associated with ESBL production (Banerjee and Johnson, 2014; Nicolas-Chanoine et al., 2014). The detection of *E. coli* ST131 in all specimens of P1 was pivotal information for the assessment of AC in this patient. Hence, fecal and saliva samples may be used to detect antimicrobial resistance agents for appropriate treatment.

CTX-M-27 and CTX-M-14 genes detected in this study belong to the group of CTX-M-9 (Bonnet, 2004), which is the predominate type in Japan, especially in nosocomial infections (Hawkey and Jones, 2009; Matsumura et al., 2015; Shibasaki et al., 2016). When any infections occur in the hospital, the analysis of CTX-M gene is important for the detection of the bacterial origin and route of transmission. We were unable to identify whether the cases were nosocomial infections because they were all emergency surgery cases. However, both P1 and P4 possibly carried ESBL-producing *E. coli*.

In this study, we retrospectively performed metagenome analysis on bile, feces, and saliva samples from six AC patients. This approach has been applied to various fields and is expected to be one of the most important diagnostic techniques to treat AC. Indeed, cases of AC where surgical intervention is undecided, the treatment process can be rapidly determined by metagenome analysis of bile collected by percutaneous transhepatic gallbladder aspiration. Hence, NGS technology provides a useful approach for the rapid diagnosis of patients with AC.

## Author contributions

MnK, MkK, and KA designed the study concept, revised the manuscript regarding intellectual content, and analyzed the data. TyS and KK provided technical support. MW, HM, TmS, and TI collected the clinical specimens from patients with AC. NK, YS, and SK approved the final draft of this manuscript to be published.

## Funding

This study was supported by a Research Program on Emerging and Re-emerging Infectious Diseases from the Japan Agency for Medical Research and Development (grant numbers: 16fk0108119j0001 and 16fk0108305j0003). The funders had no role in the study design, data collection and analysis, decision to publish, or preparation of the manuscript. This study was also supported by JSPS KAKENHI Grant Number 15K10198 and Toho University Project Research Grant Number 27-18.

### Conflict of interest statement

The authors declare that the research was conducted in the absence of any commercial or financial relationships that could be construed as a potential conflict of interest.

## Abbreviations

AC: Acute cholecystitis
ERCP: Endoscopic retrograde cholangiopancreatography
ESBLs: Extended-spectrum ß-lactamase
IBD: Inflammatory bowel disease
MLST: Multilocus sequence typing
NA: Not applicable
NGS: Next-generation sequencing
TG13: Tokyo guidelines 2013.
